# Effectiveness of Intranasal Analgesia in the Emergency Department

**DOI:** 10.3390/medicina59101746

**Published:** 2023-09-29

**Authors:** Christian Zanza, Francesco Saglietti, Jacopo Davide Giamello, Gabriele Savioli, Davide Maria Biancone, Mario Giosuè Balzanelli, Benedetta Giordano, Anna Chiara Trompeo, Yaroslava Longhitano

**Affiliations:** 1 Italian Society of Prehospital Emergency Medicine-SIS 118-Taranto, 74121 Taranto, Italy; 2 Post Graduate School of Geriatric Medicine, University of Rome “Tor Vergata”, 00133 Rome, Italy; 3 Department of Anesthesia and Critical Care, Santa Croce and Carle Hospital, 12100 Cuneo, Italy; 4 Emergency Department, Santa Croce and Carle Hospital, 12100 Cuneo, Italy; 5 Emergency Medicine and Surgery, IRCCS Fondazione Policlinico San Matteo, 27100 Pavia, Italy; 6 Department of Sensory Organs, Sapienza University of Rome, 00185 Roma, Italy; 7 Department of Human Neuroscience, Sapienza University of Rome, 00185 Roma, Italy; 8 Department of Anesthesia and Critical Care, AOU Città della Scienza e della Salute, 10126 Torino, Italy; 9 Department of Anesthesiology and Perioperative Medicine, University of Pittsburgh Medical Center, Pittsburgh, PA 15260, USA; 10 Department of Emergency Medicine, Humanitas University, 20089 Milan, Italy

**Keywords:** intranasal administration, emergency department, migraine, primary headache disorder, analgesics, acute pain management, pain, ketamine, fentanyl, paracetamol, ketorolac, nsaid

## Abstract

In the Emergency Department (ED), pain is one of the symptoms that are most frequently reported, making it one of the most significant issues for the emergency physician, but it is frequently under-treated. Intravenous (IV), oral (PO), and intramuscular (IM) delivery are the standard methods for administering acute pain relief. Firstly, we compared the safety and efficacy of IN analgesia to other conventional routes of analgesia to assess if IN analgesia may be an alternative for the management of acute pain in ED. Secondly, we analyzed the incidence and severity of adverse events (AEs) and rescue analgesia required. We performed a narrative review-based keywords in Pubmed/Medline, Scopus, EMBASE, the Cochrane Library, and Controlled Trials Register, finding only twenty randomized Clinical trials eligible in the timeline 1992–2022. A total of 2098 patients were analyzed and compared to intravenous analgesia, showing no statistical difference in adverse effects. In addition, intranasal analgesia also has a rapid onset and quick absorption. Fentanyl and ketamine are two intranasal drugs that appear promising and may be taken simply and safely while providing effective pain relief. Intravenous is simple to administer, non-invasive, rapid onset, and quick absorption; it might be a viable choice in a variety of situations to reduce patient suffering or delays in pain management.

## 1. Introduction

Acute pain is one of the most frequent symptoms in patients presenting to the Emergency Department (ED), deriving from various conditions, such as trauma, injuries, headache, renal colic, cancer, etc.

Due to its heterogeneity, it frequently represents a challenge for emergency physicians, and data indicate that inadequate pain management is rather typical [1,2].

There are many medications that can be administered, moreover using different routes. The qualities needed in the drugs we want to provide in emergency care are quickness, effectiveness, and safety. The most common routes for acute analgesia are per os (PO), intravenous (IV), and intramuscular (IM). In patients who need quick analgesia or who may have a nihil per os condition, the per os (PO) route may not be the simplest to use. However, even the intravenous administration may not be very convenient because it requires the placement of a peripheral catheter; therefore, the patient must have good venous patrimony, and the hospital requires qualified personnel.

On the other hand, IM administration frequently causes the patient discomfort. Additionally, the medication takes longer to absorb via muscle, delaying the beginning of the analgesic action.

In this scenario, because of its safety, the simplicity of administration, the non-invasive route, the quick effect, and the fact that it does not require a peripheral catheter, the intranasal (IN) method has become increasingly popular [3]. Currently, it is regarded as a good substitute for the classic methods of drug administration. Additionally, the nasal mucosa is highly vascularized and rich in capillaries; this results in a more rapid absorption and an early onset of analgesia [3,4]. Even this route of administration may have restrictions, for example, in facial trauma, bleeding nose, or white mucus.

The primary goal of the study was to compare the differences in pain scores between IN analgesics and active comparator or placebo from baseline to the time specified in the RCT. Adverse event (AE), frequency and severity, as well as the need for rescue analgesia (if available), were secondary outcomes.

There is little published material regarding opioid IN administration. Due to their difficulty in taking oral or intravenous drugs, most of the studies were conducted on pediatric patients [5,6]. Few studies have been performed on adults.

To our knowledge, there is only one review that takes a comprehensive look at the use of intranasal analgesia in emergency care and includes a small number of trials [6].

Probably, the lack of studies is due to the poor habit of using drugs with this route, few devices to use, and poor staff training. In recent years, literature about the effectiveness of intranasal analgesia has increased.

## 2. Materials and Methods

We conducted a literature review on the main databases, such as PubMed, the Cochrane Library, Medline, Embase, and Scopus in the timeline 1992–2022, using the keywords: emergency department, intranasal administration, analgesics, migraine, acute pain (MeSH Terms).

We selected all the Randomized controlled trials (RCTs) published in English and evaluated the use of IN analgesia in Emergency Care.

Only adult patients who had received at least one dosage of IN analgesia for acute pain in the Emergency Room and Prehospital Care were the only ones selected for these studies.

**Figure 1:**
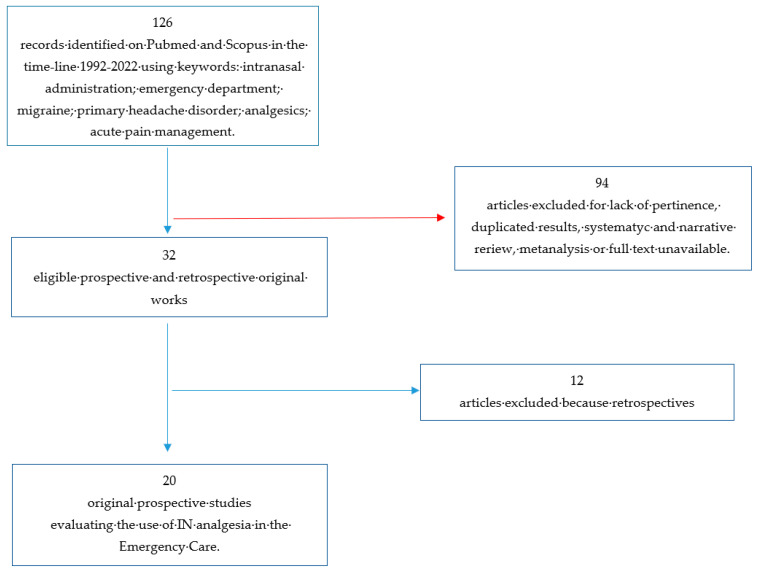
-FLOWCHART of analyzed studies.

The primary search found 126 results. Study protocols, duplicated results, not pertinent articles, and unavailability of full text were excluded. Finally, 20 clinical trials were included in this review (Figure 1), and they were evaluated for the risk of bias assessment. (Table 1 and Figure 1).

In order to assess the effectiveness and impact of the analgesics, validated pain scales were utilized in every trial that was looked at. The frequency of AEs and the requirement for rescue analgesia were also considered by these scales. The inclusion criteria of the patients participating in the trials were as follows: only adults were selected, including patients receiving IN analgesia compared to OS, IV, or a combination of different routes. Pregnant patients were not allowed. Patients were also excluded if they had used analgesics within the previous hours or if they had hemodynamic or respiratory instability or disorientation. Patients who could not provide informed consent (due to clinical impossibilities or the language barrier) were also excluded. Even patients who reported an allergy or an intolerance to the trial drug were ineligible.

The sample needed for each RCT was calculated to obtain 80% or 90% statistical power.

## 3. Results

The results are summarized in Table 2. Below, we list the main pathologies responsible for acute pain management in the ED and the implications of intranasal analgesia in these areas with side effects.

### 3.1. Headache

As known, triptans are frequently used to treat acute migraine; among all, sumatriptan is one of the most used. The oral formulation is the most prescribed, although it has several limitations, such as absorption variation and onset differences that influence the efficacy. Triptans also have well-known side effects that may restrict their effectiveness and tolerability. Few studies have investigated the use of intranasal analgesia for migraine or headache in ED [7,18,27].

A study conducted by Meredith et al. (2003) involved acute migraine. They examined the pain relief using the Visual Analogue Scale (VAS) after the administration of IV Ketorolac versus nasal sumatriptan. The study found that while both medications decreased migraine-related pain, IV Ketorolac was more efficient. However, this study has several limitations due to the limited sample size and the lack of AEs recorded [8].

Another trial examined the potential for IN Lidocaine to treat migraine; however, it was no more successful than placebo, even in addition to IV Metoclopramide (Avcu et al., 2017). The second outcome of the trial was the requirement for rescue medication, which was IV Fentanyl. Patient pain severity was assessed using the Numeric Rating Scale (NRS). Nevertheless, local discomfort brought on by lidocaine may be a confounding factor in the patient’s perceived outcome [9].

Benish et al. released the THINK Trial in 2019 with the aim of comparing the analgesia with IV metoclopramide and diphenhydramine vs. IN ketamine among patients with primary headaches in ED. All 56 of the patients they enrolled were adults. The VAS scale was used to assess changes in pain, and the results showed that standard medication was not superior to ketamine in the recruited patients. However, this RCT had several limitations. For instance, it was a single-blind trial, and patients in the control arm could have received IV Ketorolac or Dexamethasone in addition to the standard therapy, which could have influenced the comparison of analgesic effectiveness.

Savari et al. (2022) compared IN ketamine to IV ketorolac; the group treated with IN ketamine had a greater reduction in pain intensity, but they also had more adverse effects such as fatigue, dizziness, discomfort, nausea, and hypertension [11].

### 3.2. Trauma and Injuries

One of the most frequent causes of pain in ED patients is trauma, which frequently requires a combination of IM and/or IV drugs. Recently, there has been an increase in the use of IN analgesics [12].

Shimonovic et al. (2016) compared IN ketamine to IV or IM morphine; despite ketamine has been well studied as an analgesic agent, the IN administration has recently been introduced in ED. Instead, there is a lack of knowledge on the use of morphine in the literature. They enrolled a sample of 90 patients and randomized them into three groups; all three groups showed a similar level of pain relief. The study shows that IN ketamine can be used as an analgesic in emergencies since it demonstrated efficacy and safety comparable to IV and IM Morphine, and no severe AEs were noted [13].

Blancher et al. (2019) compared IN sufentanil versus IV morphine, assessing NRS at 30 min, with 4 h follow-up: they found some severe respiratory AEs (reporting a number needed to harm = 17), questioning the safety of this medication [14].

Chew et al. (2017), in a small open-label study, compared IN fentanyl added to IV tramadol and metoclopramide, showing an improvement in VAS score at 10 min, with transient side effects such as lowering in blood pressure and dizziness [15].

In 2019, performed a clinical trial on adult patients with isolated limb injuries, Lemoel et al. [16] examined two analgesic strategies: the usual treatment with IV analgesics, including opioids as rescue, versus a single dose of IV Sufentanyl followed by IV multimodal analgesia. The second approach improved pain relief after 30 min without experiencing any serious AEs, and the need for opioids or IV analgesia decreased. The majority of AEs were mild and temporary; nevertheless, they discovered a significantly high rate of respiratory events when compared to prior studies. This is likely due to ongoing monitoring of the vital signs, which may have detected events without a clinical correlation.

Tongbua et al. (2022) recently showed non-inferiority of IN ketamine compared to IV morphine for acute musculoskeletal pain in the elderly, with a quick and sustained effect (up to 120 min), without a significant difference in AEs [17].

### 3.3. Renal Colic

In the Emergency Department, renal colic is a common cause of abdominal discomfort that frequently requires a combination of analgesics, such as non-steroidal anti-inflammatory drugs and opiates; one of the most used is ketamine. In fact, numerous randomized trials have compared IN therapies to IV analgesia [28].

Farnia et al. (2017) compared IN ketamine to IV morphine, observing a statistically significant reduction in pain score, although the small sample size suggested the need for larger studies [29].

This conclusion was also supported by the study conducted by Pouraghaei et al. in 2021, which examined these two drugs showing comparable pain relief efficacy in renal colic and no relevant adverse effect [22].

In another study, Desmopressin was suggested as an alternative to the most often-used medication. The study compared Indomethacin alone versus Indomethacin with IN Desmopressin for the management of renal colic pain. However, Jalili et al. did not find IN Desmopressin to be more efficient than Indomethacin alone when compared to IN Ketamine [19].

In a comparison between IN Ketamine and IV Fentanyl, Mozafari et al. (2020) discovered that ketamine was less efficient than fentanyl and was more likely to cause side effects [20].

Nazemian et al. (2020) compared IN to IV fentanyl added to IV ketorolac. They found IN fentanyl effective in pain control, though significantly less than IV fentanyl; they concluded that this option could be considered in situations where obtaining an IV route could lead to a delay in pain control, such as overcrowded ED [21].

### 3.4. Other Situations

#### 3.4.1. Prehospital

To our knowledge, two RCTs have assessed IN analgesia in a prehospital setting.

Rickard et al. (2007) compared IN fentanyl to IV morphine, demonstrating a similar VAS reduction without a significant difference in AEs; a limitation of this trial was the lack of blindings [23].

Andolfatto et al. (2019) compared IN ketamine to a placebo when added to standard care (Nitrous Oxide), finding an improvement in pain control without severe AEs [24].

#### 3.4.2. Breakthrough Cancer Pain

A high percentage of cancer patients experience physical pain, which is frequently a chronic discomfort that ranges from moderate to severe. Breakthrough pain is a term used to describe the exacerbations of this type of pain that often occur in patients who are already receiving analgesic treatment, including opioids.

Considering the challenges associated with getting a venous route in cancer patients, IN analgesia may be helpful in treating cancer patient’s pain.

Unfortunately, there are few clinical studies of analgesic therapy for cancer patients in ED.

Only one small non-inferiority open-label RCT (Banala et al., 2020) compared IN fentanyl to IV hydromorphone in patients presenting at the ED with severe breakthrough cancer pain. Two out of three evaluations recommended the use of IN Fentanyl, which also has the benefit of requiring less time to administer. However, due to a protocol deviation (calculated by the researchers presuming that the active arm and the control one were comparable) and lack of blinding, this trial was conducted without knowing the actual pain score at baseline. This RCT might have an important bias [25].

#### 3.4.3. Acute Pain (Back and Abdominal Pain)

Sin et al. (2019) compared IN sufentanil to IV morphine in the treatment of abdominal and low back pain, finding equal improvement in NRS and AEs; the study’s limitations include a small sample size and lack of data on IV morphine rescue analgesia [26].

## 4. Discussion

Pain control is universally considered an important issue, especially in ED patients; because pain affects a patient’s quality of life, it is crucial to obtain the right treatment. Despite the availability of multimodal medications, analgesic therapy is frequently insufficient [30].

IN fentanyl and ketamine have already shown their safety in pharmacokinetics trials. These drugs have a higher bioavailability thanks to their fast absorption via the nasal mucosa and the lack of first-pass effect. It is important to remember IN route restrictions like pathologic changes to the nostrils and a limited amount of administrable volume [4,31,32].

Although there are limited trials available in the ED context, the potential benefits of IN therapies, such as rapid and simple administrations with prompt absorption, may improve pain control in ED and prehospital settings. Subdissociative doses of ketamine were proven to be safe and helpful in patients out-of-hospital, too, by a retrospective large-sample trial [33].

Low-dosage ketamine analgesia in patients with severe acute pain is becoming more and more promising due to its analgesic efficacy (similar to opioids), the potential to maintain circulatory stability and respiratory reflexes, and neuroprotection in patients with acute brain damage [34,35,36].

Opioids are being used extensively to treat acute pain, although they can have side effects that vary on dosage, including weakness, dizziness, nausea, and constipation. Due to this, the importance of multimodal analgesia with opioid-sparing techniques should be considered, especially considering the worrying data about opioid abusers. Ketamine appears to be useful in lowering the demand for opioids [37,38]. Due to its sympathomimetic action, the most significant Ketamine contraindication is coronary illness or cardiological pathologies [39].

IN route may help for severe pain requiring quick management (such as trauma or breakthrough cancer pain). The bioavailability of IN opioids depends on the specific molecule, being rather high for fentanyl and sufentanil, thanks to their lipophilic structure [40,41,42].

Regarding the safety profile, most AEs recorded were moderate and did not need medical intervention. According to the literature, dizziness was the most common. Confusion, a brief drop in blood pressure, nausea, and vomiting were also usual AEs. A small percentage of patients receiving opiate-based treatment also occasionally experienced transient bradypnea or oxygen saturation below 90%, necessitating a short-time administration of oxygen therapy. Only a minor number of serious AEs were observed in a few trials (with a small sample size).

For patients presenting with headaches, IN ketamine was found to be more effective than IV ketorolac despite a higher prevalence of side effects [7,8,9,10].

When compared to IV morphine for the treatment of pain due to traumatic injuries, IN ketamine demonstrated a non-inferiority effect; in fact, ketamine has a morphine-sparing effect. Early IN Sufentanyl administration (after triage) can enhance pain management and reduce the need for IV analgesia. For this, adding IN Fentanyl to IV Tramadol resulted in a higher decrease in pain score after 10 min. Only one research reported some significant AEs when IN Sufentanyl was compared to IV Morphine [13,17]. However, Ketamine, Fentanyl, and Morphine are thought to be the finest analgesics, per data found in the literature. The fastest onset is achieved with Ketamine and Fentanyl. Similar results on the therapy of traumatic pain were reported by Abu et al. in their review [30].

In small research, IN ketamine was found to be more effective than IV Morphine for patients with renal colic; another trial revealed that IN ketamine and IV morphine were equally beneficial. Although IN fentanyl was proven to be less efficient than IV fentanyl, it may still be used when placing a venous catheter, which could be difficult (such as overcrowding or lack of trained healthcare providers) [19,20,21,22,29].

Given that prehospital treatment is sometimes provided by paramedics, who are only trained to administer certain medications, IN analgesia may aid in the quick delivery of pain relief in this scenario. There is not much research regarding this in the literature, but in this case, IN fentanyl was found to be just as effective as IV morphine, while IN ketamine led to the fastest onset in pain relief [23,24]. A recent review (Fernandez et al., 2021) reaffirmed the safety of ketamine used outside of hospitals, with a limited rate of AEs [33].

## 5. Conclusions

These findings suggest that it might be a viable choice in a variety of situations to reduce patient suffering or delays in pain management or when accessing an intravenous route may be challenging due to clinical circumstances or a shortage of qualified healthcare professionals. In particular, in busy EDs, a standardized protocol for early analgesic administration may aid in pain treatment.

Analgesia needs to be tailored to each patient’s features, type of pain, and clinical environment. IN Fentanyl and Ketamine look promising and may be administered easily and safely while providing effective pain relief.

For this review, we could not find randomized trials including patients presenting with alteration of mental status and respiratory or hemodynamic instability, probably because of the potential impact of narcotics on breath or arterial blood pressure; none of the studies included pregnant women.

This could be a major limitation, considering that in Emergency Care, pain and respiratory or hemodynamic instability often coexist.

The absence of follow-up in this evaluation (just one research included a follow-up at 48–72 h) makes it impossible to determine if these patients need additional medications in the hours or days that followed or the frequency of subsequent adverse events (AEs). The fact that patient recruitment was completed only when researchers were available and without knowledge of the full population and the characteristics of patients who presented with pain to the ED raises the possibility of bias.

Some of the trials included were conducted on a small sample. There were few studies from Europe and Oceania; most trials were conducted in Asia or America, and no trials were undertaken in Africa. Only a small number of RCTs from Iran included patients who were 15 years old or older, whereas all the other studies included participants who were 18 years old or older. The risk of local irritation or side effects, as well as the comparison of the IN medication to placebo with a difference in pain control following the injection of analgesic or saline solution, were other possible sources of bias. In some cases (such as breakthrough cancer pain), only open-label trials were found [43].

Moreover, an optimal rescue therapy or analgesia could help hospitals out with the never-ending issue of overcrowding [44,45].

## Figures and Tables

**Table 1:** Assessment bias of analyzed studies.

First Author	Randomization Process	Deviation from Intended Intervention	Missing Outcome Data	Measurement of the Outcome	Selection of the Reported Results	Overall
Dodick et al. [7]	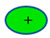	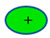	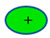	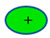	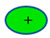	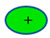
Meredith et al. [8]	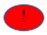	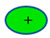	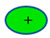	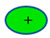	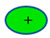	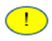
Avcu et al. [9]	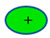	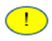	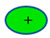	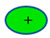	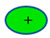	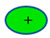
Benish et al. [10]	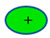	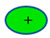	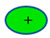	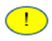	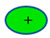	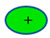
Sarvari et al. [11]	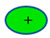	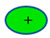	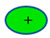	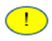	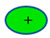	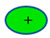
Shrestha et al. [12]	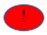	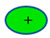	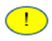	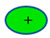	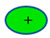	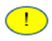
Shimonovic et al. [13]	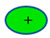	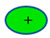	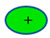	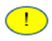	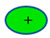	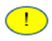
Blancher et al. [14]	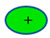	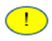	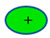	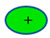	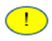	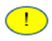
Chew et al. [15]	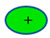	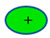	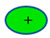	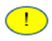	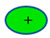	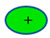
Leomoel et al. [16]	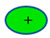	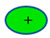	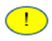	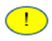	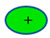	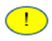
Tongbual et al. [17]	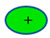	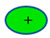	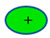	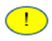	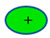	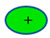
Silberstein et al. [18]	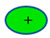	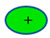	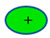	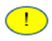	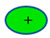	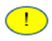
Pouraghaei et al. [19]	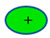	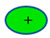	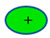	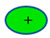	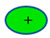	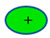
Jalili et al. [20]	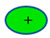	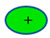	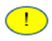	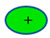	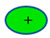	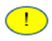
Mozafari et al. [21]	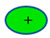	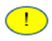	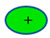	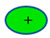	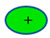	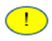
Nazemian et al. [22]	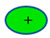	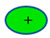	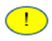	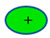	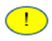	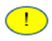
Rickard et al. [23]	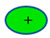	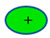	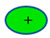	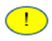	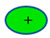	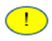
Andolfatto et al. [24]	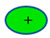	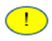	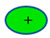	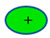	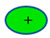	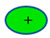
Banala et al. [25]	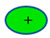	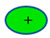	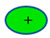	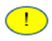	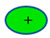	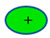
Sin et al. [26]	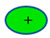	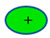	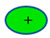	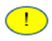	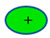	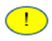

**Table 2:** Description of the study analyzed with adverse events.

Author	Intervention	Population	Objective	Findings	Adverse Events
Dodick et al. 2005 [7]	IN zolmitriptan for headache	886 zolmitriptan, 854 placebo	Headache reduction at 15 min, 30 min, 1 h, 2 h	Response rate superior in zolmitriptan (66,2%) vs. placebo (35,0%) *p* < 0.001	Adverse events (dysgeusia and nasal irritation overall) more frequent in the Zolmitriptan group
Meredith et al. 2003 [8]	IN zolmitriptan vs. IV ketorolac for headache	16 sumatriptan, 13 ketorolac	Headache reduction at 1 h	Both achieved significant pain reduction; however, ketorolac was superior in reducing VAS	Not reported
Avcu et al. 2017 [9]	IN lidocaine for headache	81 lidocaine, 81 placebo	Headache reduction at 15 and 30 min	No difference in pain reduction	Local irritation in the lidocaine group; no serious adverse reactions
Benish et al. 2019 [10]	IN ketamine vs. IV metoclopramide for headache	27 ketamine, 26 placebo	Headache reduction at 30 min and requirement for rescue at 60 min	No difference in pain reduction	No difference in the occurrence of side effects
Sarvari et al. 2022 [11]	IN ketamine vs. IV ketorolac for headache	70 ketamine, 70 ketorolac	Headache reduction at 30, 60, 120 min	Ketamine had more analgesic effect than intravenous ketorolac in a shorter time	Ketamine group reported increased incidence of dizziness, HR increase, and BP increase
Shrestha et al. 2016 [12]	Effectiveness of IN ketamine in pain reduction (various acute injuries)	39 patients	Pain reduction at 15, 30, 60 min	IN ketamine reduced VAS pain scores to a clinically significant degree in 80% of patients	Most common side effects were dizziness, nausea, and sedation
Shimonovic et al. 2016 [13]	IN ketamine vs. IV morphine vs. IM morphine in acute traumatic pain	34 IN ketamine, 26 IV morphine, 30 IM morphine	Pain at 5 min interval from 0 to 60 min	IN ketamine may provide analgesia clinically equal to IV or IM morphine	Ketamine group reported increased incidence of difficulty in concentrating, dizziness, confusion
Blancher et al. 2019 [14]	IN sufentanil vs. IV morphine in acute pain	77 IN sufentanil, 80 IV morphine	Non-inferiority study	IN sufentanil was non-inferior to IV morphine	Incidence of adverse events was higher in the IN group
Chew et al. 2017 [15]	IN fentanil plus IV tramadol vs. IV tramadol in acute pain	10 IN fent. + IV tramad., 10 IV tramadol	Pain reduction at 10 min	Greater reduction in the mean VAS score among the patients in the fentanyl + tramadol arm	Fentanyl + tramadol group had an increased incidence of sleepiness
Lemoel et al. 2019 [16]	IN sufentanil vs. IN placebo in acute pain (all plus IV multimodal analgesia)	72 IN sufentanil, 72 IN placebo	Proportion of VAS < 3 at 30 min	IN sufentanil determines a 20% absolute increase in proportion of patients reaching pain relief	IN sufentanil group showed an increased incidence of opioid-related adverse events
Tongbual et al. 2022 [17]	IN ketamine vs. IV morphine in musculoskeletal pain in ED	37 IN ketamine, 37 IN morphine	Pain reduction at 30 min	IN ketamine provides analgesic efficacy comparable (non-inferior) to IV morphine	No substantial differences in adverse effects.
Silberstein et al. 2017 [18]	Sumatriptan nasal powder (with IN delivery system) vs. oral sumatriptan in migraine	765 nasal powder, 766 oral sumatriptan	Headache reduction at 30 min	Sumatriptan powder provided greater reduction in migraine pain intensity	IN group showed an increased incidence of local adverse effects (irritation, bad taste)
Pouraghaei et al. 2021 [19]	IN ketamine vs. IV morphine in renal colic	100 IN ketamine, 100 IV morphine	Pain reduction at 15, 30, 60 min	IN ketamine has the same efficacy as IV morphine in renal colic pain control	No remarkable side effects occurred after IN ketamine
Jalili et al. 2019 [20]	Indomethacin plus IN desmopressin vs. Indomethacin plus IN placebo in renal colic	62 IN desmopressin, 62 IN placebo	Pain reduction	Desmopressin, as an adjunct to NSAIDs in the management of renal colic, does not significantly improve pain relief	No severe adverse event (e.g., chest pain, anaphylaxis, and dyspnea) occurred for any of the patients
Mozafari et al. 2020 [21]	IN ketamine vs. IV fentanil in renal colic	65 IN ketamine, 65 IV fentanil	Pain reduction at 5, 15, 30 min	The effect of IN ketamine was less significant than of IV fentanil	No difference in the occurrence of side effects (more common nausea, vomiting, dizziness)
Nazemian et al. 2020 [22]	IN fentanil plus IV ketorolac vs. IV fentanil plus IV ketorolac in renal colic	110 IN fentanil, 110 IV fentanil	Pain reduction at 60 min	The mean pain score was higher in the IN group. Nevertheless, the pain intensity significantly and consecutively reduced in both groups during the study	IV fentanil: nausea and pruritus; IN fentanil: bad taste and pharyngeal irritation
Rickard et al. 2007 [23]	IV morphine vs. IN fentanil in prehospital analgesia	122 IV morphine, 136 IN fentanil	Difference between baseline and destination pain score	No difference in pain reduction	IN fentanyl group showed an increased incidence of opioid-related events
Andolfatto et al. 2019 [24]	Effectiveness of IN ketamine in pain reduction in prehospital setting	60 IN ketamine, 60 IN placebo	Pain reduction at 2 and 30 min	Intranasal ketamine provides clinically significant pain reduction and improved comfort compared with intranasal placebo	Adverse events were more frequent in ketamine group: unreality, dizziness, nausea, fatigue
Banala et al. 2020 [25]	IN fentanil vs. IV hydromorphone in cancer pain in ED setting	42 IN fentanil, 42 IV hydromorphone	Pain reduction at 60 min	Two of three analyses supported non-inferiority of INF versus IVH, while one analysis was inconclusive	Not reported
Sin et al. 2019 [26]	IN sufentanil vs. IV morphine in acute pain in ED	30 IN sufentanil, 30 IV morphine	Efficacy and safety of IN sufentanil in ED	IN resulted in safe analgesia, comparable with IV morphine	There were no significant differences in the incidence of adverse events between the groups

## Data Availability

Not applicable.
